# *Mycoplasma genitalium* and *M. pneumoniae* Regulate a Distinct Set of Protein-Coding Genes in Epithelial Cells

**DOI:** 10.3389/fimmu.2021.738431

**Published:** 2021-10-11

**Authors:** Enrique I. Ramos, Kishore Das, Alana L. Harrison, Anissa Garcia, Shrikanth S. Gadad, Subramanian Dhandayuthapani

**Affiliations:** ^1^ Center of Emphasis in Cancer, Department of Molecular and Translational Medicine, Texas Tech University Health Sciences Center, El Paso, TX, United States; ^2^ Center of Emphasis in Infectious Diseases, Department of Molecular and Translational Medicine, Texas Tech University Health Sciences Center El Paso, El Paso, TX, United States; ^3^ Graduate School of Biomedical Sciences, Texas Tech University Health Sciences Center El Paso, El Paso, TX, United States; ^4^ Mays Cancer Center, UT Health San Antonio MD Anderson Cancer Center, San Antonio, TX, United States

**Keywords:** mycoplasma, epithelial cells, interactions, RNA-seq, gene expression, protein-coding

## Abstract

*Mycoplasma genitalium* and *M. pneumoniae* are two significant mycoplasmas that infect the urogenital and respiratory tracts of humans. Despite distinct tissue tropisms, they both have similar pathogenic mechanisms and infect/invade epithelial cells in the respective regions and persist within these cells. However, the pathogenic mechanisms of these species in terms of bacterium-host interactions are poorly understood. To gain insights on this, we infected HeLa cells independently with *M. genitalium* and *M. pneumoniae* and assessed gene expression by whole transcriptome sequencing (RNA-seq) approach. The results revealed that HeLa cells respond to *M. genitalium* and *M. pneumoniae* differently by regulating various protein-coding genes. Though there is a significant overlap between the genes regulated by these species, many of the differentially expressed genes were specific to each species. KEGG pathway and signaling network analyses revealed that the genes specific to *M. genitalium* are more related to cellular processes. In contrast, the genes specific to *M. pneumoniae* infection are correlated with immune response and inflammation, possibly suggesting that *M. pneumoniae* has some inherent ability to modulate host immune pathways.

## Introduction

The genus mycoplasma contains unique bacterial species that lack ‘cell wall’, a characteristic feature of the bacteria. They are believed to have originated from gram-positive bacteria by ‘reductive evolution’ or ‘losing genes’ (1). Consequently, mycoplasmas become the smallest self-replicating organisms with genome sizes ranging from 500-1350 Kb. Although most mycoplasmas lead a parasitic or commensal lifestyle and are found associated with other organisms, some mycoplasmas are serious pathogens and cause diseases in humans, cattle, fish, and plants (2). *Mycoplasma genitalium* and *Mycoplasma pneumoniae* are two significant mycoplasmas infecting humans. Whereas *M. genitalium* is associated with non-gonococcal urethritis in males and cervicitis in females (3–5), *M. pneumoniae* is implicated in a range of respiratory diseases such as atypical pneumonia, asthma, bronchitis, and others (1, 6). *M. genitalium* is also implicated in women’s reproductive tract maladies such as pelvic inflammatory disease and infertility (7, 8).

Although *M. genitalium and M. pneumoniae* have distinct tissue tropisms, they show strikingly similar pathogenic mechanisms (9, 10). They both infect and invade the urogenital tract and respiratory tract epithelial cells, respectively, and persist within these cells (10–12). As in many bacteria, *M. genitalium* and *M. pneumoniae* attach with the epithelial cells to initiate colonization. Interestingly, the attachment of these pathogens to host cells is mediated by an attachment organelle located at the tip of the flask-shaped bacteria (9, 10). The attachment organelle involves or has a bunch of proteins known as adhesins and adherence-related accessory proteins. While adhesins localized at the tip of the organelle bind to the host cells, adherence-related proteins help form the tip organelle and position the adhesins at the tip. Studies have demonstrated that these proteins have significant homologies and identities between these two species. For example, the major adhesin P140 (MG191) of *M. genitalium* has been reported to be a homolog of *M. pneumoniae* adhesin P1 (MPN141) (13) and both P140 and P1 exhibit extensive immunological cross-reactivities (14). Similarly, the adherence related proteins P32 (MG318) (15), HMW1 (MG312), HMW2 (MG218), and HMW3 (MG317) are considered homologs of *M. pneumoniae* P30 (MPN453), HMW1 (MPN447), HMW2 (MPN310), and HMW3 (MPN452) (16). It is also reported that the genes encoding adhesins and adherence-related proteins are clustered in three different loci in the genomes of both *M. genitalium* and *M. pneumoniae* (17). Intriguingly, *M. genitalium* or *M. pneumoniae* deficient in either adhesion or adherence related proteins, lack the attachment organelle and, as a consequence, fail to attach with eukaryotic cells and even lose motility (16, 18–20), suggesting that both adhesins, as well as adherence related proteins, are critical for the virulence and pathogenesis of these species.

Besides, both *M. genitalium* and *M. pneumoniae* similarly interact with host cells, and they cause cytotoxicity to epithelial cells (21, 22). Although the precise mechanism of cytotoxicity remains unclear, hydrogen peroxide released by these bacteria is believed to induce cytotoxicity (6, 23). However, it has been reported that the cytotoxicity caused by *M. genitalium* depends on the adhesion of the bacterium to the host cells (24). Further, despite the absence of cell walls, both pathogens interact with TLR receptors, mainly TLR1, 2, and 6, on the host cells and induce proinflammatory cytokines by activating NF-кB (12, 25). Interaction with TLR is presumed to be mediated by lipid-associated membrane proteins (LAMPs) located abundantly on the surface of these pathogens. Genome sequences reveal that *M. genitalium* and *M. pneumoniae* possess twenty-one genes and forty-six genes, respectively, to encode LAMPs (26). The direct involvement of LAMPs in the induction of immune response in the host cells has also been demonstrated (25, 27, 28). While You et al. (28) have shown that total purified LAMPs from *M. genitalium* induce cytokines in human monocytic cells, Shimizu et al. (27) have reported that LAMPs derived from *M. pneumoniae* induce NF-кB in monocytic cells. Also, an *M. genitalium* LAMP protein MG309 is noticed to induce proinflammatory cytokines in human genital epithelial cells by binding with TLR2 and TLR6 receptors (25).

Although significant advances have been made over the past years in understanding *M. genitalium*/*M. pneumoniae-host* interactions, there are several areas for which information is still lacking. Particularly, our understanding of how *M. genitalium* and *M. pneumoniae* invade host cells, persist within the epithelial cells or evade host immune responses is very poor. When bacteria infect host cells, the cells respond to infection by differentially expressing their genes. The differentially expressed genes (DEG) are very likely to provide information on pathways altered by bacterial infection and, ultimately, the modulation of host cells by bacteria. In recent years, RNA-seq has become a standard tool to analyze gene expression in eukaryotic and bacterial cells (29, 30). RNA-seq is a next-generation sequencing tool that identifies and quantifies the expressed transcripts (31, 32). It also doesn’t require any sequencing primers or pre-established oligonucleotide arrays (33). Thus, in this study, we employed an RNA-seq based transcriptomic approach to understanding the DEG with *M. genitalium* or *M. pneumoniae* infection. Here we report that *M. genitalium* and *M. pneumoniae* dysregulate the expression of a distinct set of protein-coding genes in HeLa cells, suggesting that the pathogenic mechanisms of these pathogens may be different from each other.

## Materials and Methods

### HeLa Cell Culture

HeLa cells (CCL-2) were cultured in Dulbecco’s modified Eagle’s medium (DMEM) with 10% FBS in a humid chamber at 37°C and 5% CO_2_. After confluent growth, cells were washed twice with 1x sterile PBS, trypsinized, and counted in an automatic cell counter. After counting, the cells were plated (1.5x10^6^ cells/well) in a 6-well culture plate and grown for 24 h before infection. To minimize variations, we avoided passaging HeLa cells cultures and always used stock cultures passaged only once after obtaining from ATCC.

### Mycoplasma Culture

*M. genitalium* G37 (ATCC #33530) and *M. pneumoniae* M129 (ATCC #29343) were grown in 100 ml of SP-4 medium at 37°C in 150 cm^2^ tissue culture flasks (Corning, NY) for three to four days or until the color of the medium changes to light orange. The adherent mycoplasmas are scraped from the culture flasks, washed with 1x sterile PBS, and suspended in PBS. The bacterial suspension was passed through a 23G needle to disperse the clumps and diluted to obtain OD_600 =_ 1.00, equivalent to 1X 10^7^ CFU/ml. This was further diluted with PBS to the required multiplicity of infection.

### Isolation OF RNA

HeLa cells (1.5x10^6^ cells/well) in each well of 6-well culture plate were infected with *M. genitalium* G37 or *M. pneumoniae* at an MOI of 1:10 (1 cell: 10 bacteria) or uninfected (control cells) and incubated for 4 h at 37°C in a CO_2_ incubator. This MOI and incubation time were based on our preliminary microscopic analysis. At this condition, only minimal or no necrosis/cell death of Mg or Mp infected HeLa cells was observed; hence we thought this condition was ideal for comparing the gene expression altered by these species. After the incubation, the cells were collected, washed with PBS, and RNA isolated using EZ-10 DNAaway RNA Mini-Preps Kit (Bio Basic, Toronto, Canada). Agilent Technologies 4200 TapeStation was used to analyze RNA integrity of the preparation, and RNA quantification was done using Nanodrop. 4ug of total RNA with >9 RIN (RNA Integrity Number) was used for RNA-seq library preparation.

### RNA-Seq Library Preparation and Sequencing

RNA-sequencing libraries were prepared and sequenced at Novogene Corporation Inc. (Sacramento, CA, USA). After sample preparation, sample quality control was carried out by Nanodrop, agarose gel electrophoresis, and Agilent 2100. After the QC procedures, mRNA from eukaryotic organisms was enriched using oligo (dT) beads. First, the mRNA is fragmented randomly by adding a fragmentation buffer. Then the cDNA is synthesized by using an mRNA template and random hexamers primer, after which a custom second-strand synthesis buffer (Illumina), dNTPs, RNase H, and DNA polymerase I are added to initiate the second-strand synthesis. Second, after a series of steps, including terminal repair, A ligation, and sequencing adaptor ligation, the double-stranded cDNA library is completed through size selection and PCR enrichment. Quality control of the library consists of three steps: Quibit 2.0, Agilent 2100, and Q-PCR. Finally, qualified libraries are fed into Illumina sequencers (NovaSeq 6000) after pooling according to their effective concentration and expected data volume.

### Quality Control Metrics

QC metrics at different timepoints throughout the analysis were applied to ensure good standards (**Data Sheet 1**; **Supplementary Figures 1, 2**). Raw fastq sequencing reads quality was assessed using the bioinformatic program FastQC (Babraham Bioinformatics). The average quality per read was 36 (Phred score) (**Data Sheet 1**; **Supplementary Figure 1**).

The quality of the transcriptome assembly was evaluated using the Hisat2 alignment metrics, FeatureCounts assignment metrics, and the bioinformatics program MultiQC (34). **Data Sheet 1**; **Supplementary Table 1** shows the alignment statistics for all the biological samples.

Clustering of the sequencing samples was carried out employing Principal Component Analysis using the R statistical software (version 4.0.3) with DESeq2 package applying the function plotPCA() for each condition (**Data Sheet 1**; **Supplementary Figures 2B, 3B**).

Gene expression quality was measured by using the MA plot and volcano plot. To this end, the MA plot was generated using the R package DESeq2 calling the function plotMA() (**Data Sheet 1**; **Supplementary Figures 2C, 3C**), while the volcano plot was generated using the evaluate an expression in a data environment function (with()) (**Data Sheet 1**; **Supplementary Figures 2D, 3D**). The settings for the volcano plot include plotting log2FoldChange, -log10(p-value), and highlighting specific points that pass the padj<0.05.

**Figure 1:**
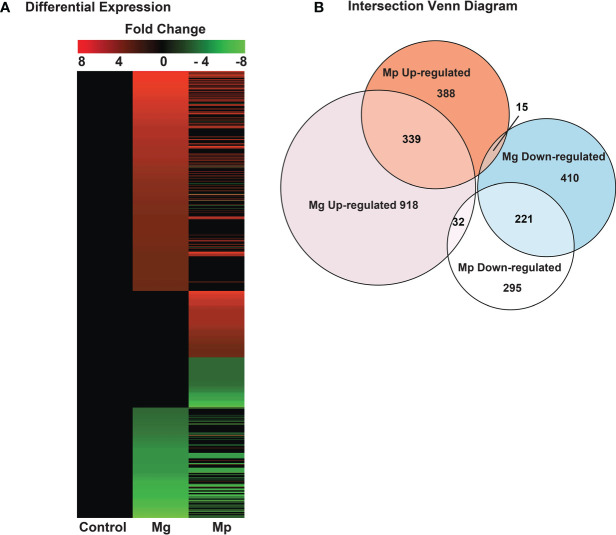
Profile of RNA-seq based differentially expressed genes in *M. genitalium* (Mg) and *M. pneumoniae* (Mp). **(A)** Heat map from the entire differentially expressed genes dataset. Only differentially expressed genes with 1.5-fold change were considered for subsequent analyses. **(B)** Intersection Venn diagram of differentially expressed genes in Mg and Mp samples.

Differentially expressed genes obtained from DESeq and **Figure 1A** were normalized using scaled TPM (transcripts per million) values obtained from the bioinformatic program StringTie gene abundance output. The heat map was plotted using R statistical software with the R package pheatmap. (**Data Sheet 1**; **Supplementary Figure 4**).

### Transcriptome Assembly and Differential Expression

Sequencing and mapping of NGS paired-end reads (150 bp) to the human transcriptome (hg38) with annotation from Ensembl release version 101 (August 2020) using the HISAT2 aligner (35). SAM files were converted into BAM files, then sorted and indexed using Samtools (36). Gene abundance output was generated using standard settings by employing the bioinformatics program StringTie (37). Read counts were computed using the alignment files and the FeatureCounts program from the subread-2.0.1 package (38) with reference annotation from Ensembl version 101. In the initial annotation, a total of 60,670 genomic features are considered. Counts normalization and differential gene expression analyses were calculated using the DESeq2 R package (39) and edgeR (40). Input files for DESeq2 are a metadata file with normal/experimental conditions and the read counts obtained from FeatureCounts, and two replicates were used for each condition. Settings for DESeq2 DE analysis include: study design=~ 0+Condition, rowSums(cpms)>0, estimateSizeFactors(dds), counts(sf, normalized=T), log2(norm), DESeq(dds, parallel=FALSE), results(dds, contrast = c(“Condition”, “Experimental”, “Normal”), format=‘DataFrame, pAdjustMethod =‘BH’, independentFiltering = FALSE, parallel=FALSE). Filtering criteria for differentially expressed genes of interest were only protein-coding genes obtained from the R package biomaRt annotation querying Ensembl database and a DESeq2 log2FoldChange greater than 1.5-fold change. The heat map was generated in R statistical software (version 4.0.3) using the package plotly.

### Gene Ontology and KEGG Pathways

Biotype classification and composition analyses were done using the Ensembl database (REST API) and biomaRt R package (41) on filtered differentially expressed genes. Gene Ontology (GO) was carried out by querying the bioinformatic Database for Annotation, Visualization, and Integrated Discovery (DAVID) (42). The gene list of interest was uploaded and queried on the online database using the Ensembl Gene ID identifier and selecting the list type as a gene list. The following annotation categories were included in the analysis: KEGG Pathways, GO Biological Processes, and UCSC TFBS. Settings for the functional annotation charts include a threshold count of 2, EASE score 0.1, Display Benjamini, and # of Records 1000. The results file was downloaded (**Data Sheets 2–14**; **Supplementary Files**) and parsed using R statistical software (version 4.0.3); subsequently, the data was visualized and plotted using the R packages ggplot2 and plotly for dot plots and heatmaps, respectively. Normalized Enrichment Scores (NES) were calculated using fgsea (Bioconductor) (43) package for fast pre-ranked gene set enrichment analysis (GSEA). Different gene datasets were obtained from the Molecular Signatures Database (MSigDB) (https://www.gsea-msigdb.org) to query genes in the pathway (**Data Sheets 2–14**; **Supplementary Files**): Hallmark Pathways, KEGG Pathways, Molecular functions, and Biological processes. Settings for the analysis include a number of permutations = 1000, and stats = ranks. Heat maps were generated using plotly (R package) using the Normalized Enrichment Score (NES).

### Protein-Protein Interaction Network Analysis

Network interaction for protein-coding genes was generated using the bioinformatic database STRING-DB (44) version (11.0b, October 2020 release). Default settings were used to query STRING-DB. The confidence score cutoff was set to 0.40. The number of indirect interactions was enhanced to obtain a complete pathway analysis (10-40 additional indirect interactions). Network visualization was done using the open-source software Cytoscape (version 3.8.2) (45), expression data (log2FoldChange) obtained from the study was included in the network using the Style/Node/Fill Color settings, as well as the edge weight, was set to the STRING-DB confidence score. Enrichment of gene ontology was added to the network analysis to supplement using the Reactome FI (46) database and identify potential relationships between differentially expressed genes and biological processes. The Cluster FI Network algorithm was applied to the network, and different modules/clusters were generated. The settings for the Reactome FI were set to standard settings using an FDR filter of 0.05, then top pathways from each module were identified and annotated.

## Results

### *M. genitalium* and *M. pneumoniae* Differentially Regulate Host Genes

To understand host genes regulated by human pathogenic mycoplasmas, gene expression analyses in HeLa cells infected, independently, with *M. genitalium* (Mg) and *M. pneumoniae* (Mp) were performed by directional PolyA+ RNA-seq as detailed in the methods section. A heat map (**Figure 1A** and **Data Sheet 1**; **Supplementary Figure 4**) of the expressed protein-coding genes revealed that several genes in infected cells are differentially expressed compared to non-infected cells. Also, it showed significant qualitative and quantitative differences in gene expression between the Mg and Mp infected cells. In cells infected with Mg, a total of 1289 and 646 genes were up-and downregulated, respectively, in relation to non-infected cells. A similar trend was also seen with the HeLa cells infected with Mp but with a relatively lower number of up-regulated (742) and downregulated (548) genes (**Figure 1B**). To gain better insights on this, the number of genes altered or affected by each mycoplasma species was analyzed by an intersectional Venn diagram (**Figure 1B**). This indicated that many genes, 339 of the up-regulated and 221 of the downregulated genes, are commonly targeted by both mycoplasmas. Simultaneously, this displayed that each mycoplasma specifically targets several host genes. The pathogen Mg specifically up-and downregulated the expression of 918 and 410 genes, respectively, while Mp specifically up-and downregulated the expression of 388 and 295 genes. A breakdown of all the regulated genes in Mg and Mp infected cells is shown in **Supplementary Files**; **Data Sheet 11**. The gene showing the highest expression in Mg-infected cells was found to be the gene associated with chromosome 2 of humans and encoding the protein C2orf72, for which the function is currently unknown.

Conversely, the highest up-regulated gene in Mp infected cells was noticed to be the gene encoding the protein PECAM-1 or CD31, a protein associated with cell adhesion. Nonetheless, the highest up-regulated gene in both Mg and Mp infected cells appears to be the gene encoding the protein PPFIA4, a protein related to the disassembly of cell adhesion. Thus, overall, gene expression analyses of mycoplasma infected cells indicate that Mg and Mp target some common and species-specific genes in the host cells.

### *M. genitalium* and *M. pneumoniae* Regulate Distinct Host Pathways

**Figure 2:**
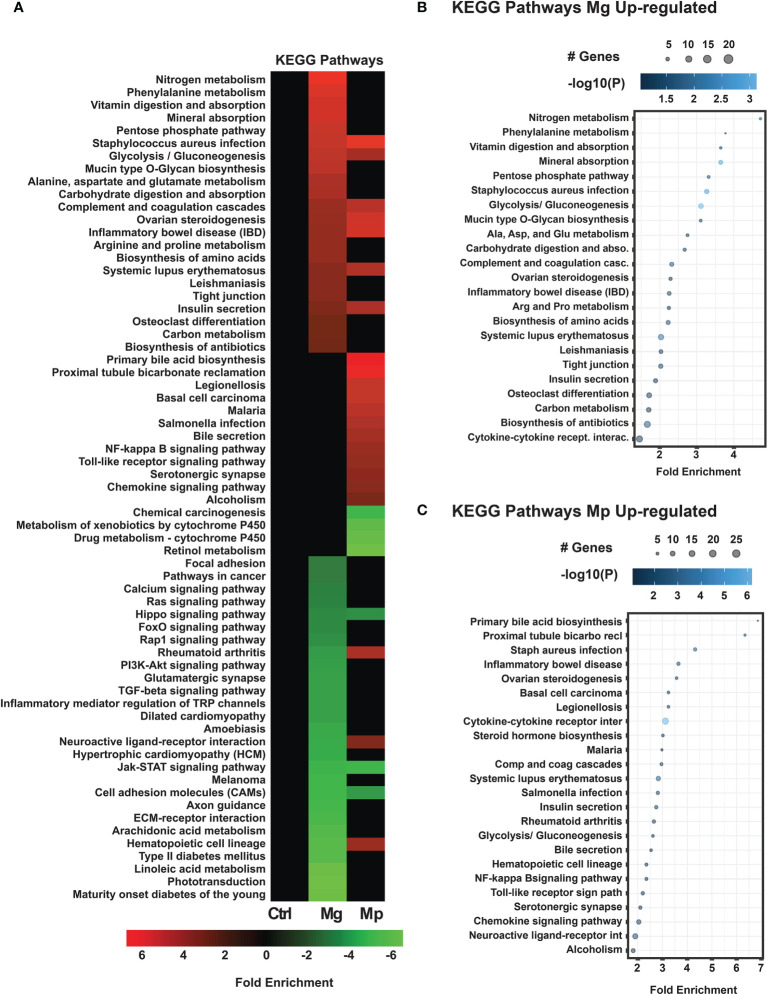
KEGG Pathways enrichment analysis for *M. genitalium* (Mg) and *M. pneumoniae* (Mp) regulated genes. **(A)** KEGG pathways heat map showing fold enrichment contrast between Mg and Mp. **(B)** Dot blot for Mg up-regulated genes, **(C)** Dot blot for Mp up-regulated genes. Dot blots measure fold enrichment of the pathways, dot size represents total number of genes included in each category, and gradient color shows statistical significance [-log_10_(*P*)].

Further to understand the pathways regulated or affected by Mg and Mp in the host cells, we performed KEGG pathways enrichment analysis of the differentially expressed host genes. Data shown in **Figure 2A** demonstrates that Mg and Mp target distinct pathways in host cells, although there are some overlaps (**Data Sheet 1**; **Supplementary Table 3**). The up-regulated genes with Mg infection show enrichment of genes predominantly for pathways related to nitrogen metabolism, vitamin digestion and absorption, mineral absorption, and pentose phosphate utilization (**Figure 2B**). Additionally, enrichment of genes for pathways related to inflammation and infection such as *Staphylococcus aureus* infection, inflammatory bowel disease (IBD), leishmaniasis infection, and tight junction are also noted. Interestingly, up-regulated genes upon Mp infection also show enrichment of genes for the latter pathways (**Figure 2C**). However, this encompasses a large number of other pathways related to infection and inflammation. Some notable pathways of this category are legionella infection, salmonella infection, malaria infection, NF-кB, Toll-like receptor signaling, cytokine, cytokine receptor interaction, chemokine signaling, and others (**Figure 2C**). Possibly this may suggest that Mp has more ability to alter the pathways related to the immune response in the host than Mg.

**Figure 3:**
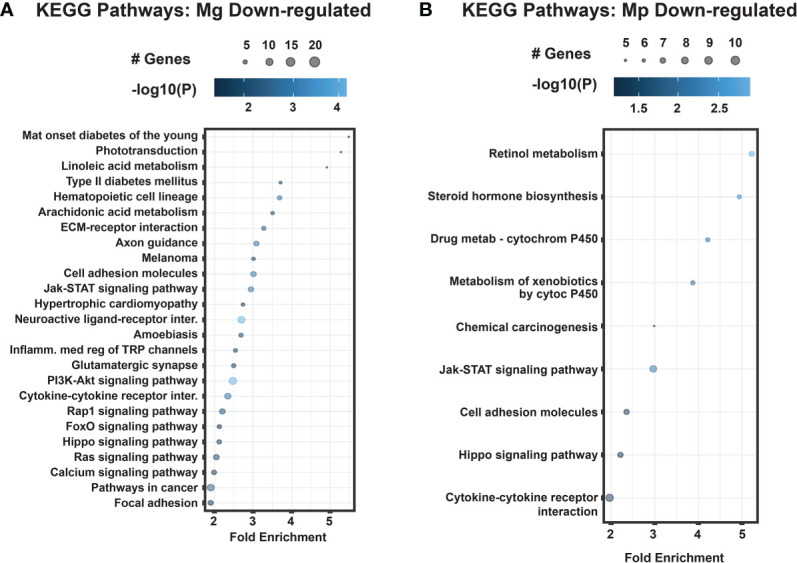
KEGG pathways for differentially expressed down-regulated genes *M. genitalium* (Mg) and *M. pneumoniae* (Mp). **(A)** Dot blot for Mg down-regulated genes, **(B)** Dot blot for Mp down-regulated genes. Dot blots measure fold enrichment of the pathways, dot size represents total number of genes included in each category, and gradient color shows statistical significance [-log_10_(*P*)].

With regard to downregulated genes, both Mg and Mp have significant enrichment for Jak-STAT signaling, Hippo signaling, and cell adhesion pathways in the host cells (**Figures 3A, B**). In addition, however, the downregulated genes by Mg are highly enriched for critical signaling pathways such as Ras, Fox, PI3K-Akt, Rap, chemokine receptors, and TGF-β. This is very significant because these pathways are not affected by genes regulated by Mp infection.

### *M. genitalium* and *M. pneumoniae* Target Distinct Host Biological Processes

**Figure 4:**
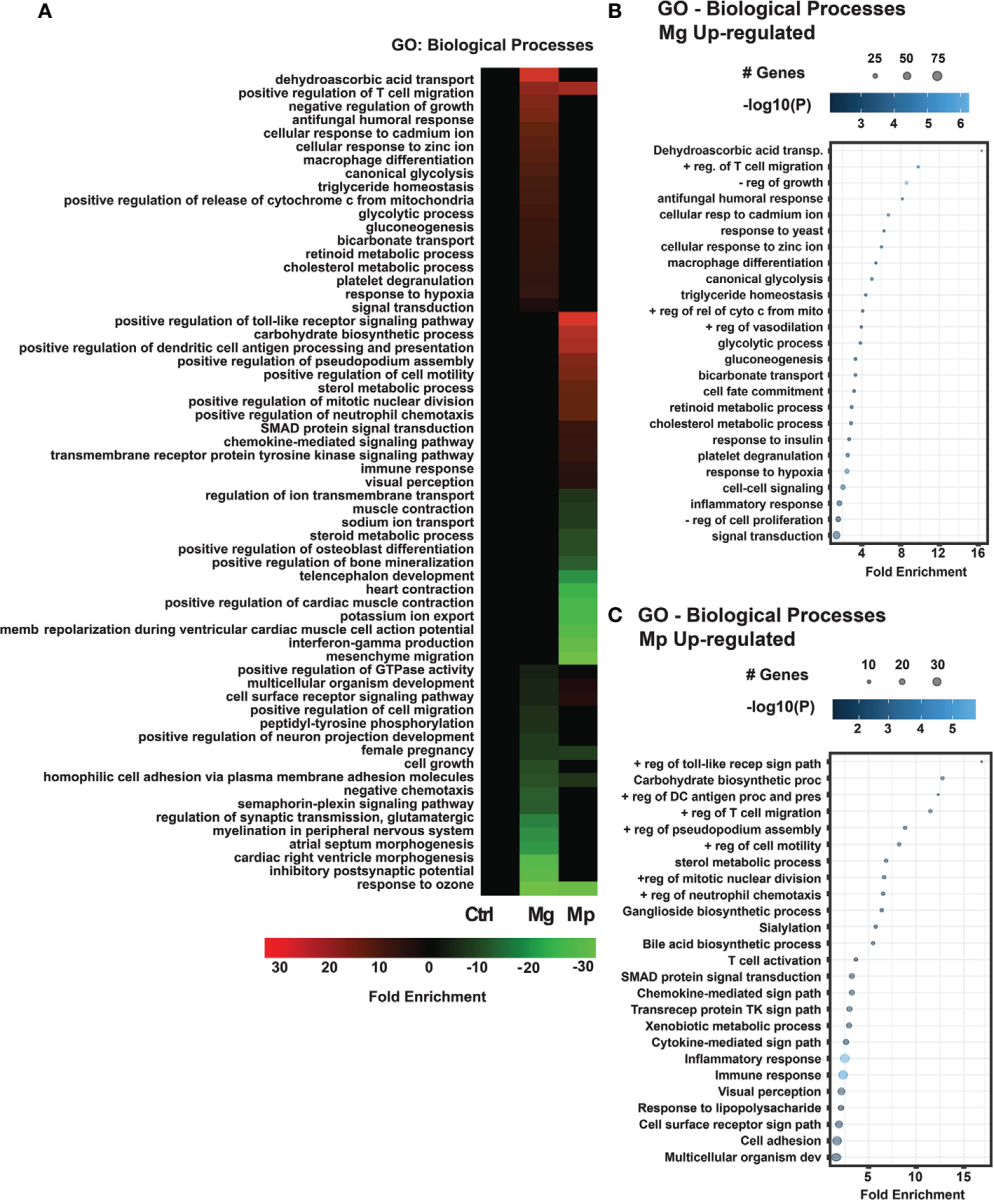
GO Biological processes enrichment analysis for *M. genitalium* (Mg) and *M. pneumoniae* (Mp). **(A)** GO Biological Processes heat map showing fold enrichment profiles in Mg and Mp. **(B)** Dot blot for the enriched biological process of differentially expressed up-regulated genes in Mg. **(C)** Dot blot for the enriched biological process of differentially expressed up-regulated genes in Mp. Dot blots measure fold enrichment of the pathways, dot size represents total number of genes included in each category, and gradient color shows statistical significance [-log_10_(*P*)].

**Figure 5:**
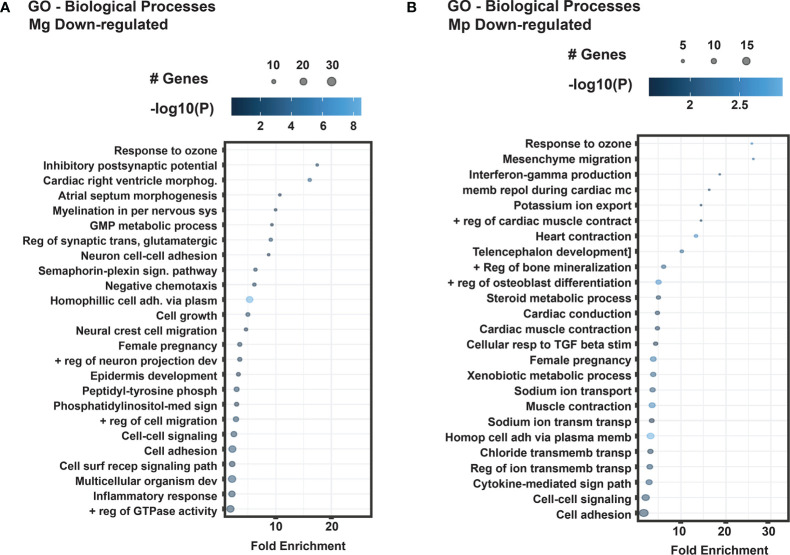
GO Biological processes of differentially expressed down-regulated genes in *M. genitalium* (Mg) and *M. pneumoniae* (Mp). **(A)** Dot blot for the enriched biological process of differentially expressed down-regulated genes in Mg. **(B)** Dot blot for the enriched biological process of differentially expressed down-regulated genes in Mp. Dot blots measure fold enrichment of the pathways, and dot size represents total number of genes included in each category, and gradient color shows statistical significance [-log_10_(*P*)].

We also analyzed how Mg and Mp infections affected the host biological processes by subjecting the differentially expressed genes to GO (Gene Ontology) analysis. As noticed for KEGG pathways, the GO analysis also showed distinct enrichment patterns for Mg and Mp regulated genes (**Figure 4A**). Biological processes related to transport (dehydroascorbic acid, bicarbonate), metal ions (cadmium, zinc), metabolism (glycolysis, gluconeogenesis, retinoid, cholesterol), macrophage differentiation, and signal transduction are the ones that show enrichment in Mg up-regulated genes (**Figure 4B**). On the contrary, enrichment of genes for biological processes such as toll-like receptor signaling, antigen processing and presentation, chemokine-mediated signaling, neutrophil chemotaxis, inflammatory response, and immune response are seen with Mp up-regulated genes suggesting that Mp primarily targets the immune process (**Figure 4C**). Besides, genes for metabolic process, namely carbohydrate biosynthetic process and sterol metabolic process (**Figure 4C**), are also enriched with Mp infection. Similar to that of up-regulated genes, the genes downregulated by Mg and Mp infection show enrichment for diverse biological processes (**Figure 5**). While genes downregulated by Mg show enrichment for several biological processes, including signaling pathways, the genes downregulated by Mp are enriched for processes associated with ion transport (sodium, potassium, chloride) (**Figure 5**). It should be noted that enrichment of genes for ion transport is noticed with up-regulated genes with Mg infection.

### *M. genitalium* and *M. pneumoniae* Target Genes Controlled by Diverse Transcription Factors

**Figure 6:**
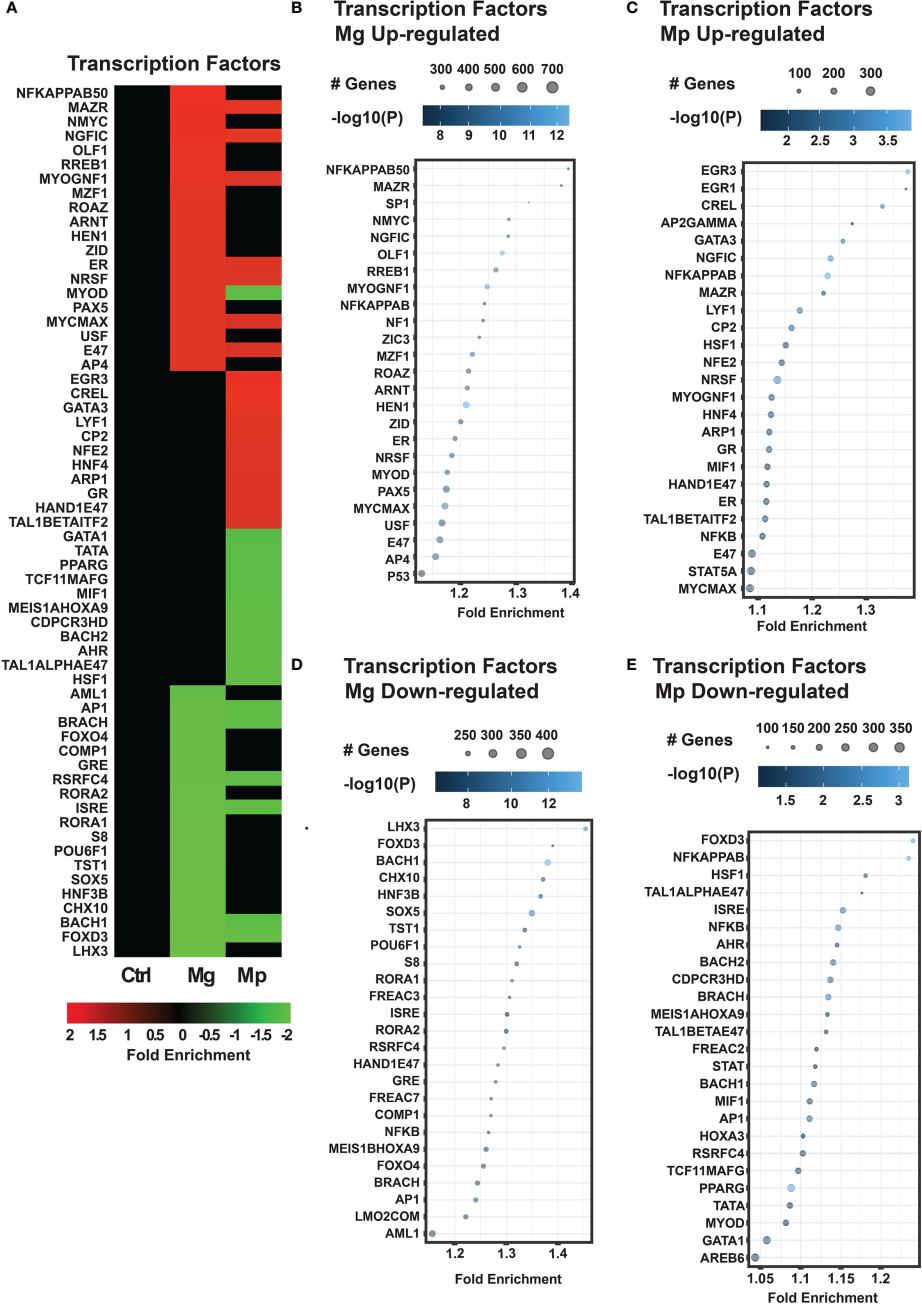
*M. genitalium* (Mg) and *M. pneumoniae* (Mp) regulate gene targets of specific transcription factors. **(A)** Transcription factor heat map showing fold enrichment profiles. Quantification of transcription factors enrichment in the RNA-seq dataset by dot blots for each condition: **(B)** Mg up-regulated, **(C)** Mp up-regulated, **(D)** Mg down-regulated, and **(E)** Mp down-regulated. Dot blots measure fold enrichment of the pathways, and dot size represents total number of genes included in each category, and gradient color shows statistical significance [-log_10_(*P*)].

We further interrogated whether the specific genes targeted by each mycoplasma are controlled by the same set of transcription factors or different transcription factors. This revealed that Mg and Mp infections target genes regulated by distinct transcription factors (**Figure 6A**). Mg shows enrichment of up-regulated genes for transcription factors such as NFKAPPAB50, MAZR, SP1, and NMYC, which govern the expression of genes involved in inflammation, cell proliferation, and immune response (**Figure 6B**). By contrast, the pathogen Mp shows enrichment of transcription factors EGR3, EGR1, CREL, AP2GAMMA, and GATA3, which control gene expression in muscle and memory development, NFKB signaling, and inflammation (**Figure 6C**). A noteworthy observation is that both Mg and Mp have enrichment of genes transcription factor NFKAPPAB50 and related factors which play a significant role in regulating immune response and inflammation during infection. Enrichment of transcription factors for the downregulated genes of both Mg and Mp also shows some specificity. The transcription factors LHX3, FOXD3, BACH1, and CHX10, which affect the expression of genes needed for early development and immunity (**Figure 6D**), are highly enriched in the Mg downregulated genes. Conversely, the transcription factors FOXD3, NFKAPPAB, and HSF1 play roles in development, metabolism, and inflammation (**Figure 6E**), enriched in the Mp downregulated genes.

### *M. genitalium* and *M. pneumoniae* Regulate Distinct Signaling Networks

**Figure 7:**
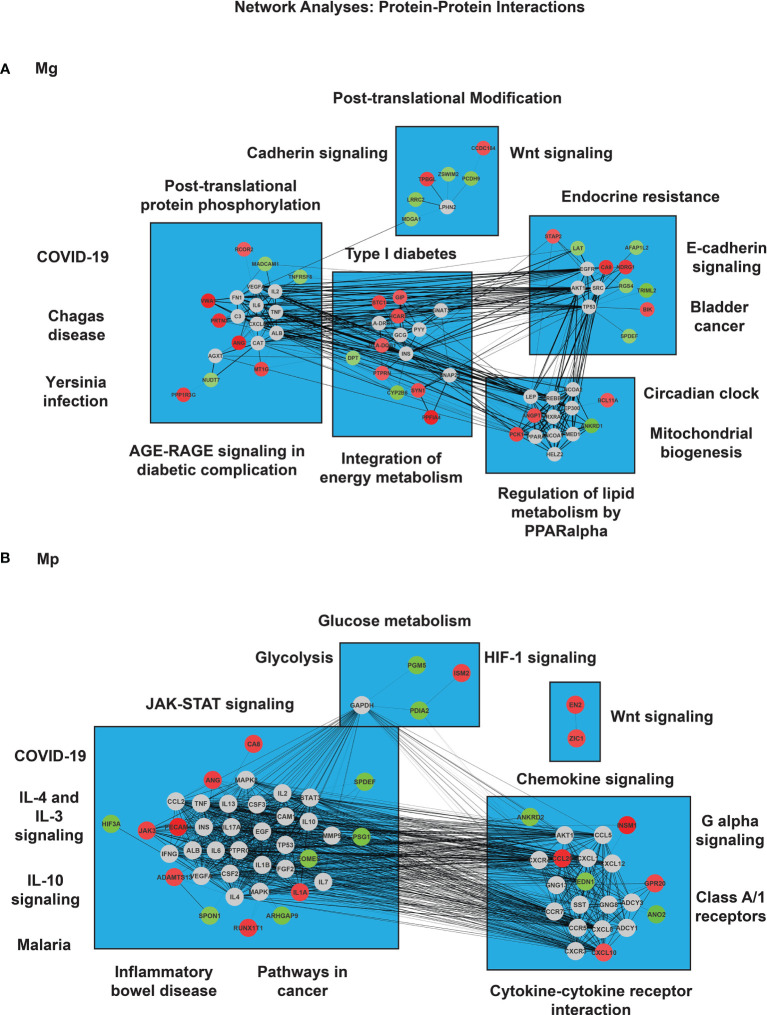
Network analysis of protein-coding genes in *M. genitalium* (Mg) and *M. pneumoniae* (Mp). To determine functional cross-talk between both mycoplasma infected conditions, a gene-network analysis was carried out. **(A)** Gene-gene interactions in Mg and **(B)** Gene-gene interactions in Mp. The colors of the node correspond to either overexpression or knock-down expression (red and green, respectively). Gray nodes correspond to indirect interactions not obtained directly from the RNA-seq dataset. The width of the edge corresponds to the degree of evidence for the predicted interaction.

Network analysis was done to determine functional cross-talk between genes regulated by each mycoplasma infection (**Figure 7**). Mg infection encompassed 67 nodes, of which 22 are associated with up-regulated or higher expression and 15 with downregulated or lower expression. Some noted regulated pathways include E-cadherin- and Wnt-signaling and protein-coding genes associated with phosphorylation (downregulation of *MDGA1*) (**Figure 7A**). Additionally, the Mg-regulated network comprised of pathways related to post-translational protein phosphorylation (*VWA1*-upregulated), Type I diabetes mellitus (*PTPRRN*- and *HLA-DQB1*-upregulated), endocrine resistance (*BIK*-upregulated), and regulation of lipid metabolism by PPAR-alpha (*ANGPTL4*-upregulated) (**Figure 7A**).

With MP infection, there were 65 nodes in which 14 were highly overexpressed, and the expression of 11 was attenuated. Some affected pathways include *IL-4* and *IL-3* signaling (*JAK3*- and *I-1A*-upregulated), *IL-10* signaling (*IL-1A*-upregulated), malaria infection (*PECAM1*-upregulated), inflammatory bowel disease (IL-1A-upregulated), pathways in cancer (*JAK3*- and *RUNX1T1*-upregulated), and JAK-STAT signaling pathway (*JAK3*-upregulated) (**Figure 7B**). Moreover, the second cluster of genes depicted altered expression in the following pathways: Chemokine signaling pathway (*CCL20*- and *CXCL10*-upregulated), G-alpha signaling (*CCL20*- and *CXCL10*-upregulated), class A/1 receptors (*EDN1*-downregulated, as well as *CCL20*- and *CXCL10*-upregulated, and cytokine-cytokine receptor interaction (*CCL20*- and *CXCL10*-upregulated). In addition, network analysis showed increased interactions between *ZIC1* and *EN2* genes with no direct interaction with additional genes from the dataset (**Figure 7B**).

Interestingly, two gene networks involved in COVID-19 infection were found to be present indirectly in the study for Mg (*CXCL8*, *TNF*, *IL-2*, *C3*, *IL-6*) and Mp (*CSF3*, *CSF2*, *TNF*, *MAPK8*, *CCL2*, *MAPK*, *STAT3*, *IL-2*, *IL-6*, and *IL-1B*) (**Figure 7**).

Overall, Mg infection led to more overexpression of protein-coding genes, affecting cellular processes to a larger extent than Mp infection.

## Discussion

Both Mg and Mp infect humans by attaching with and invading epithelial cells in the urogenital tract and respiratory tract, respectively (10–12). Therefore, this study investigated how the infection of these pathogens affects the expression of protein-coding genes in the HeLa epithelial cell line that may directly control the rate, mode, and severity of infection. Infection of both species led to differentially expressed genes (DEG) in HeLa cells, reinforcing the concept that alteration of host genes is an essential process during infection (29). Although infection by both species induced up- and downregulation of genes in HeLa cells, the number of DEG (1714 genes) with Mg-infected cells was slightly higher than those observed (1275 genes) with Mp-infected cells. The increased number of DEG with Mg infection is little surprising because the genome size of Mp is relatively more significant and has more genes to encode proteins than Mg (26). This may indicate that pathogens’ size or protein-coding ability play a minor role in host-pathogen interactions. Previously, DEG based on Genome-wide Transcriptomic Analysis of human cervical epithelial cells infected with Mg and cells from bronchoalveolar fluids of children infected with Mp have been reported (47, 48). Further, very recently, host DEG have been described for several bacterial infections, including mycoplasmas, such as *Mycobacterium leprae* (49), *Mycobacterium tuberculosis* (50), *Bordetella pertussis* (51), *Streptococcus pneumoniae* (52), *Orientia tsutsugamushi* (53), *Mycoplasma gallispeticum* (54) and *Mycoplasma ovopneumoniae* (55). However, our study is the first to directly compare genome-wide transcriptome analyses of host cells infected with Mg and Mp.

Our data reveal that Mg and Mp target completely different pathways and processes in the host cells. This may be readily discernible from the heat maps (**Figures 1**–**4**) shown for separate analyses such as fold change, KEGG, GO, and transcription factors. In line with this, the network analysis (**Figure 7**) of DEGs also shows entirely different patterns for these species. This is not only striking but also surprising because both these species hitherto have been considered to have similar pathogenic mechanisms (10, 56). In host-pathogen interactions, surface components and secreted proteins of the pathogens play significant roles in regulating the host genes and cellular processes (57–59). Thus, the observation that Mg and Mp regulate different genes in the host cells may indicate that the surface components between Mg and Mp are very different.

Furthermore, as cell wall-less bacteria, Mg and Mp lack lipopolysaccharides, lipoteichoic acids, and peptidoglycans, the conventional pathogen-associated molecular patterns (PAMPs) can interact with host-pathogen recognition receptors (PRRs) such as TLRs, C-type lectin, and other receptors. In place of conventional PAMPs, mycoplasmas lipid-associated membrane proteins (LAMPs) on their surface can interact with TLRs (60). Mp has a significantly higher number of genes (46 genes) encoding LAMPs than Mg (21 genes) (26) and it is not clear at present whether these determine the specificity of the DEGs in the host cells.

Moreover, very little knowledge exists about secretory proteins by these pathogens. Both Mg and Mp genomes show a complete absence of genes encoding traditional secretory pathways associated with the pathogenesis, such as type 3 or type 4 (26). Therefore, the possibility of bacteria-derived secreted proteins altering the host gene expression during mycoplasma infection is minimal. However, a significant factor distinguishing Mg from Mp is a toxin, CARDS (community-acquired respiratory distress syndrome) toxin, encoded by the gene *MPN372*, in Mp (61). Although this toxin has no export signals, about ten percent of this toxin has been reported on the cell surface of Mp (62). Extensive studies with *Escherichia coli* expressed and purified toxin indicated that it has a role in altering inflammatory responses (63, 64). However, whether this toxin has any role in regulating DEGs in the host cells remains to be investigated.

Further, a significant target of Mg infection in the host seems to be the proteins associated with signaling systems. This is obvious from the enrichment of genes for several signaling pathways in both up-and downregulated genes of Mg-infected cells. Interestingly, however, genes are significantly enriched for cancer-related signaling pathways such as PI3-Akt, RAS, FoxO, Rap, and HIPPO. Further, the KEGG analysis reveals that a set of genes are explicitly enriched for ‘pathways in cancer.’ This is very remarkable and, at the same time, raises the question of whether Mg has any role in inducing cancer in infected individuals. There are studies in the literature which connect Mg infection with cancer. For example, Namiki et al. (65) had demonstrated that persistent exposure of benign human prostate cells (BPH-1) to Mg transformed the cells into cancer cells. Besides, our study has indicated that Mg infection in C33-A epithelial cells can affect the expression of the oncogenic protein p53, particularly the acetylated p53 (Lys 382) (24). Acetylated p53 downregulates the expression of p21 protein, an essential protein associated with cell cycle growth arrest. Theoretically, therefore, inhibition of p53 by *M. genitalium* may have the ability to escape from growth arrest leading to transformation. As stated, this is very hypothetical, and there is every possibility that alteration of cancer-related signaling pathways by Mg may lead to some unknown consequences other than cancer. The signaling pathways always cross-talk, and the signaling pathways altered by Mg are also involved in cellular processes supporting this view.

Further, a recent study on bovine epithelial cells on Lactobacillus infection has also reported enrichment of PI3K-Akt Hippo, FoxO, and p53 signaling pathways (66). Furthermore, although a previous study (67) has examined the DEG in cervical epithelial cells infected with Mg, we are unable to relate our p53 data with this study because its focus was comparing the DEG associated with proinflammatory immune response and host defense between infected and non-infected cells. Nevertheless, it should be noted that our KEGG pathway analysis, similar to their study, revealed up-and down-regulation of genes associated with immune and inflammatory pathways.

It is also interesting that DEGs in Mp infected cells show significant enrichment pathways related to immune response, cytokines, inflammation, and other related processes. While the KEGG pathways show about fifteen genes related to cytokine-cytokine receptors, the GO analysis reveals enrichment of 20-30 genes each for immune and inflammatory responses. Although DEGs of Mg-infected cells show such enrichments, those are relatively smaller than that of DEGs of Mp infected cells, suggesting that Mp infection can induce robust immune and inflammatory responses in the host. The first and foremost evidence that Mp can cause an inflammatory response comes from its infection in humans. The Mp-associated diseases such as pharyngitis, bronchitis, pneumonia, asthma, and other chronic respiratory illnesses are due to excessive immune and inflammatory responses (68). Secondly, evidence results from experiments to support that interaction of Mp or its components with host cells induces immune or inflammatory responses (68). For instance, it has been shown that *M. pneumoniae* with protease or antibodies against P1 adhesin failed to induce proinflammatory cytokines in lung epithelial cells, indicating that adhesion of the bacterium with cells can induce an inflammatory response.

Further, purified LAMPs encoded by *M. pneumoniae* genes *MPN602, MPN162, and MPN611* induce inflammatory responses on the host cells has extensively been studied (27, 60, 69). Similarly, purified CARDS toxin encoded by the gene *MPN372* has been shown to regulate NLRP3 inflammasome activity in the host cells (70, 71). These prior observations with Mp infection justify the enrichment of DEGs towards immune and inflammatory responses. Moreover, evidence that Mp infection can cause severe inflammatory response comes from RNA-seq analysis of the bronchoalveolar fluid of Mp infected children and non-infected children (47, 48). This revealed the upregulation of several genes related to mononuclear cell proliferation and signaling. In addition, this study (47) noticed the upregulation of signaling genes associated with activation of NK and CD8+ T cells, reiterating that Mp targets mainly on altering immune response in the host.

In summary, we have demonstrated that Mg and Mp infection of HeLa cells leads to differential expression of protein-coding genes. Furthermore, the differentially expressed genes for each species are enriched for specific pathways and cellular processes, suggesting that the Mg and Mp have diverse pathogenic mechanisms. It is presumed that future RNA-seq studies using mutant strains of Mg and Mp and primary cells from humans and animals will fully unravel the differences in pathogenic mechanisms between these two species. In particular, dual RNA-seq (30, 49, 52, 53, 72), which can determine the changes in transcription in both mycoplasma pathogens and host cells, will lead to a better understanding of the pathogenic mechanisms of the species.

## Data Availability Statement

The datasets presented in this study can be found in online repositories. The names of the repository/repositories and accession number(s) can be found below: https://www.ncbi.nlm.nih.gov/, GSE179051.

## Author Contributions

Data curation, KD, ER, AG, SD, and SG. Methodology, ER. Software, ER. Investigation, KD and ER. Resources, SD and SG. Writing—original draft preparation, ER, AH, SD, and SG. Writing—review and editing, SD and SG. Supervision, SD and SG. Project administration, SD and SG. funding acquisition, SD and SG. All authors contributed to the article and approved the submitted version.

## Funding

SD is partly supported by an NIH 1R15AI156647 grant. SG is supported by a First-time Faculty Recruitment Award from the Cancer Prevention and Research Institute of Texas (CPRIT; RR170020).

## Conflict of Interest

The authors declare that the research was conducted in the absence of any commercial or financial relationships that could be construed as a potential conflict of interest.

## Publisher’s Note

All claims expressed in this article are solely those of the authors and do not necessarily represent those of their affiliated organizations, or those of the publisher, the editors and the reviewers. Any product that may be evaluated in this article, or claim that may be made by its manufacturer, is not guaranteed or endorsed by the publisher.
