# Supplementary material for: Mycoplasma genitalium and M. pneumoniae Regulate a Distinct Set of Protein-Coding Genes in Epithelial Cells
Source: Front Immunol. 2021 Oct 11;12:738431. doi: 10.3389/fimmu.2021.738431 (PMC8544821; doi:10.3389/fimmu.2021.738431)
Supplement: Supplementary file 1 [file DataSheet_1.pdf]

Supplementary Materials

"Mycoplasma genitalium and *M. pneumoniae* regulate a distinct set of protein-coding genes in epithelial cells"

Ramos *et al.* (2021)

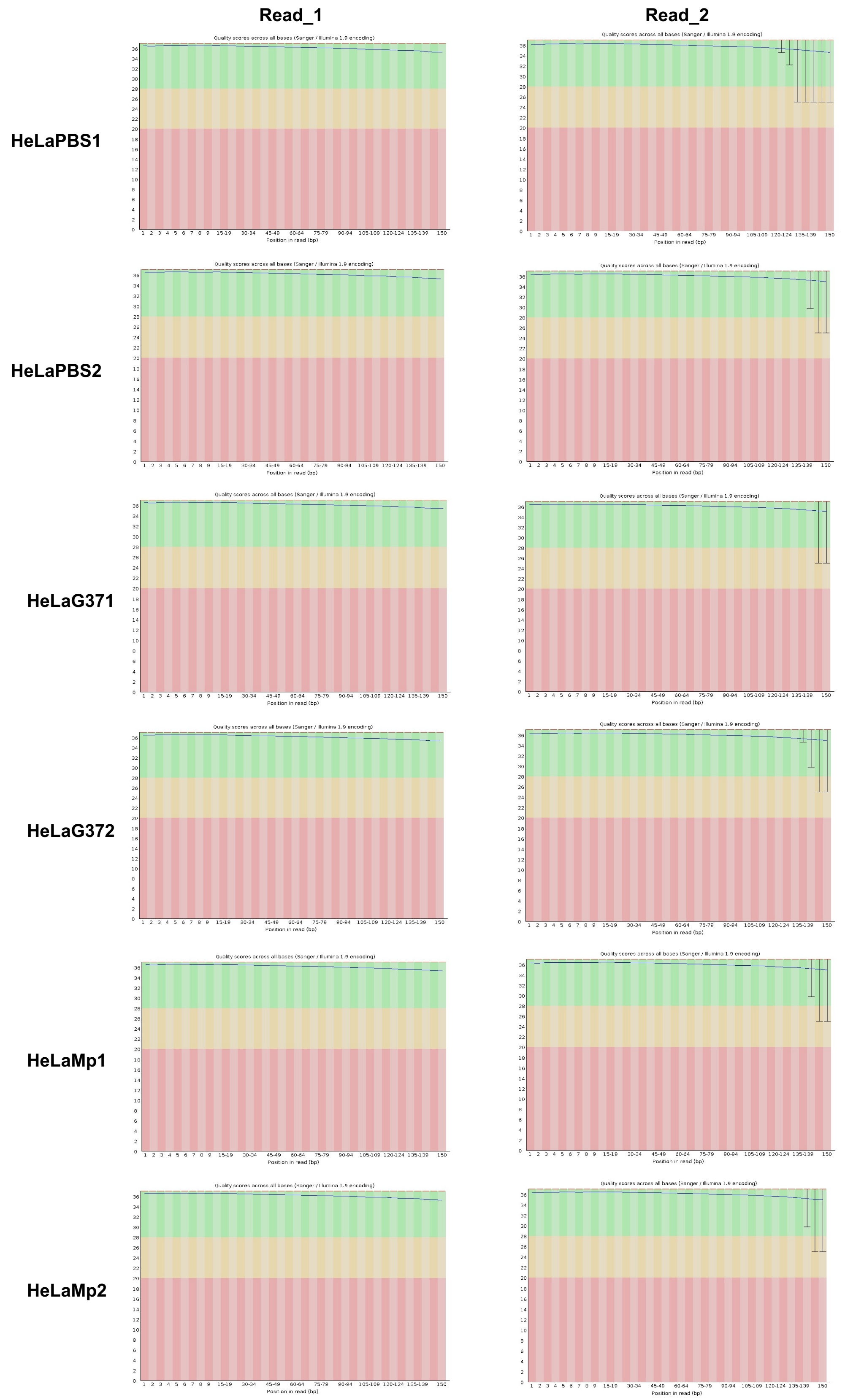

Supplementary figure 1: Reads quality metrics

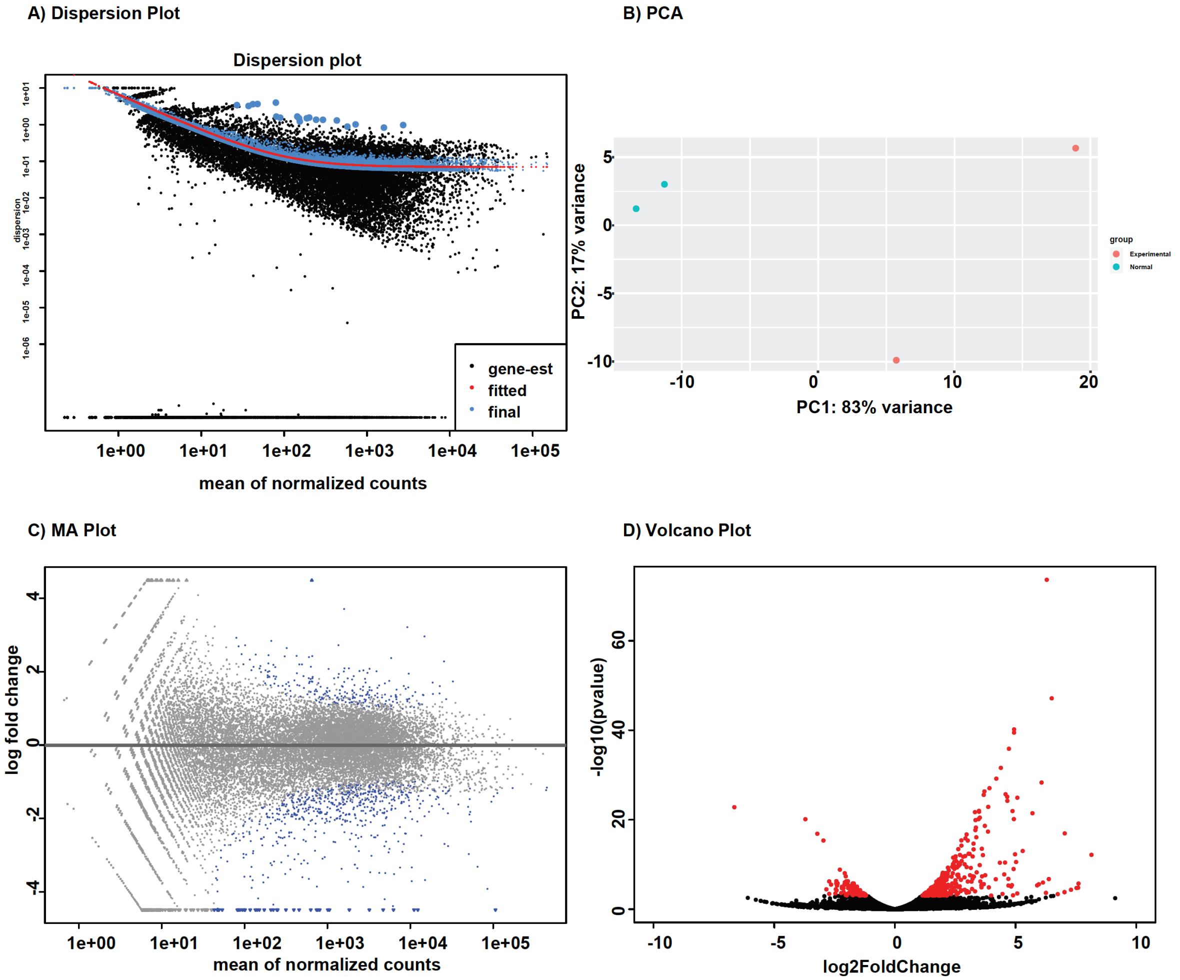

Supplementary figure 2: Mg. A. Dispersion plot: The curve is displayed (red line) to estimate the expected dispersion value for genes of a given expression strength. Each point (black dot) is a gene with an associated mean expression level and maximum likelihood estimation of the dispersion. This is a measure to ensure the data is a good fit for the DESeq2 model. The computed model shows data scatter around the curve with the dispersion decreasing with increasing mean expression levels. **B.** PCA: To assess the quality of the samples, we used a sample clustering algorithm employing Principal Component Analysis (PCA). The different colors represent the experimental (Mg) compared to normal (PBS). The x-axis represents PC1 with 83% of variance explained, while the y-axis represents PC2 with 17% variance, the replicates cluster together and are separated by a larger magnitude on the x-axis (PC1). **C.** MA plot: The MA plot visualizes the variances between measurements taken in two samples by transforming the data onto log-ratio (M) and mean average (A) scales followed by plotting the different values. The x-axis represents the mean of normalized counts, while the y-axis represents log fold change. Black points are not significant, while blue points are significant, triangles represent a data point above the axis threshold. **D.** Volcano plot: The volcano plot displays statistical significance (P-value) versus magnitude change (fold change). Allowing fast visual identification of genes with large fold changes that are also statistically significant. The x-axis represents log2 Fold Change while the y-axis -log10(p-value). Black points are not significant, while red points pass the padj <0.05 threshold.

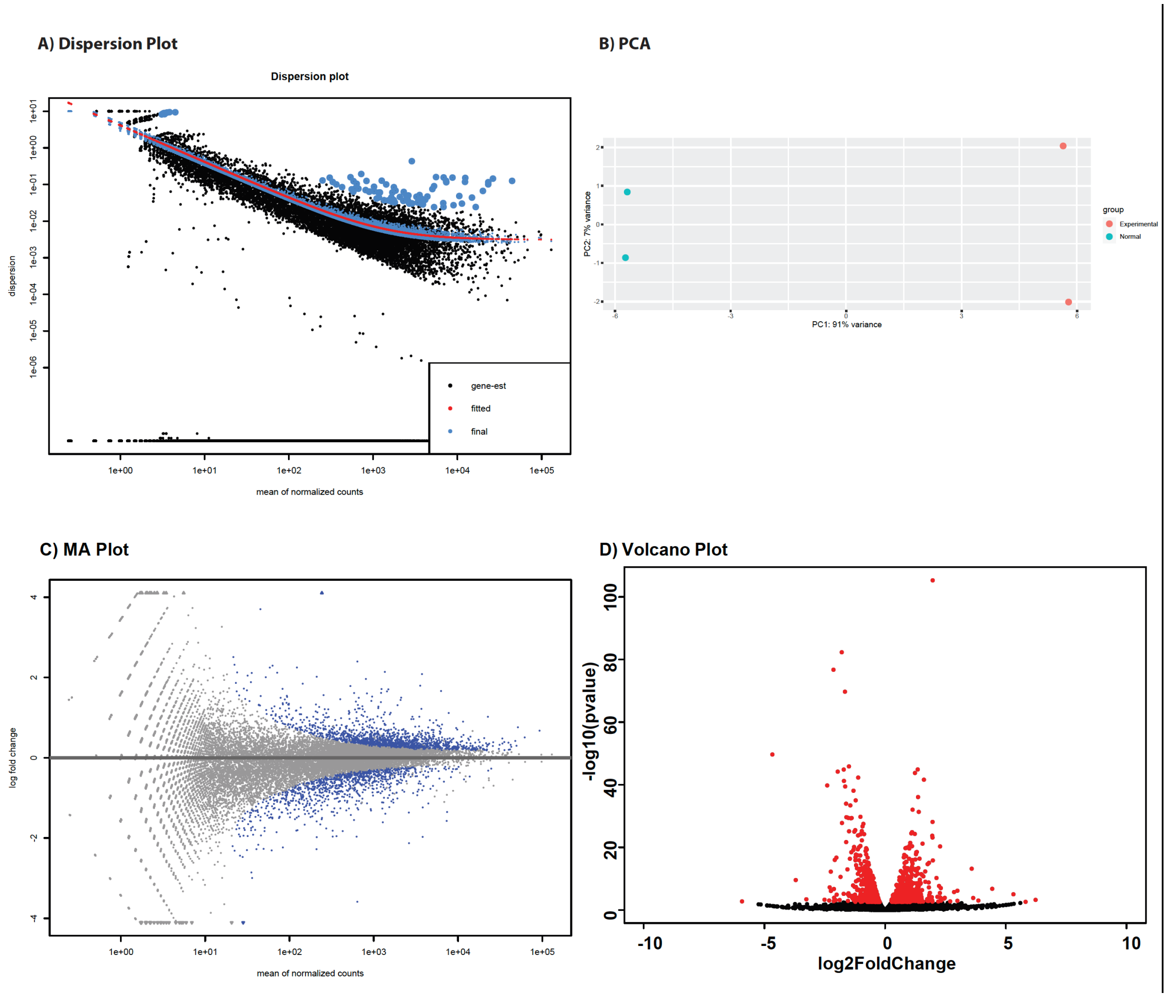

Supplementary figure 3: Mp. A. Dispersion plot: The curve is displayed (red line) to estimate the expected dispersion value for genes of a given expression strength. Each point (black dot) is a gene with an associated mean expression level and maximum likelihood estimation of the dispersion. This is a measure to ensure the data is a good fit for the DESeq2 model. The computed model shows data scatter around the curve with the dispersion decreasing with increasing mean expression levels. **B.** PCA: To assess the quality of the samples, we used a sample clustering algorithm employing Principal Component Analysis (PCA). The different colors represent the experimental (Mp) compared to normal (PBS). The x-axis represents PC1 with 83% of variance explained, while the y-axis represents PC2 with 17% variance, the replicates cluster together and are separated by a larger magnitude on the x-axis (PC1). **C.** MA plot: The MA plot visualizes the variances between measurements taken in two samples by transforming the data onto log-ratio (M) and mean average (A) scales followed by plotting the different values. The x-axis represents the mean of normalized counts, while the y-axis represents log fold change. Black points are not significant, while blue points are significant, triangles represent a data point above the axis threshold. **D.** Volcano plot: The volcano plot displays statistical significance (P-value) versus magnitude change (fold change). Allowing fast visual identification of genes with large fold changes that are also statistically significant. The x-axis represents log2 Fold Change while the y-axis -log10(p-value). Black points are not significant, while red points pass the padj <0.05 threshold.

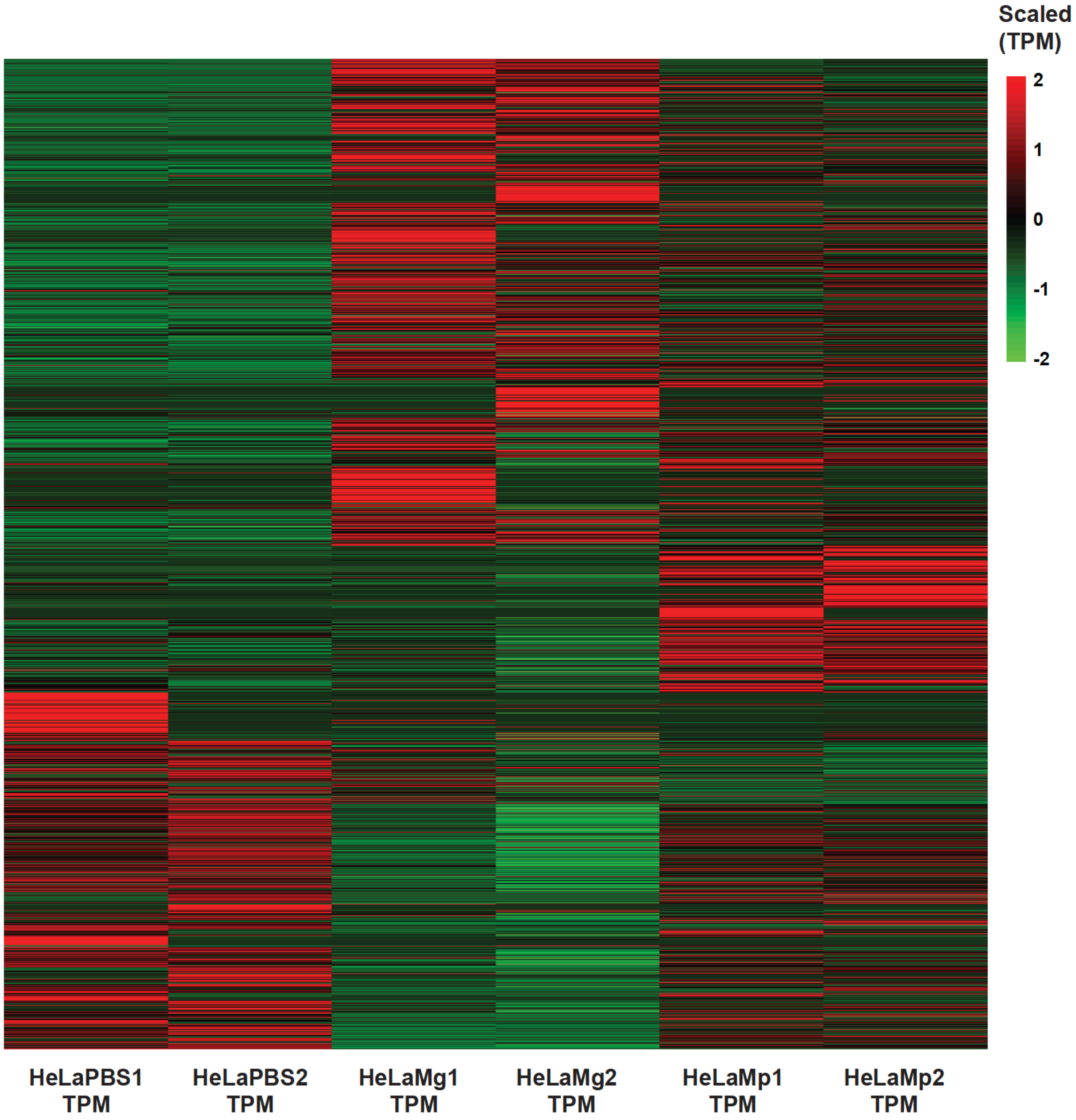

Supplementary figure 4: Representation of differentially expressed genes (DEGs) from Figure 1A based on normalized gene expression (scaled TPM – Transcripts Per Million). The total number of genes for the analysis is 2,618 genes. The red color represents up-regulation (2 scaled TPM) while the green represents down-regulation (-2 scaled TPM). TPM values were centered and scaled in the row direction.

Supplementary Table 1. Transcriptome assembly metrics.

Each sample was sequencing on Illumina NovaSeq 6000 platform. The sequence length of each read is 150 bp.

Sample	Average Quality Per Read	Total Raw Reads (PE)	Uniquely Aligned Reads (PE)	Total Aligned Reads (PE)	FeatureCounts Annotation *Homo_sapiens* GRCh38.101.gtf	Feature Counts Assignments
**HeLaPBS1**	36 Phred Score	21538559	18956287 (88%)	20478147 (95.08%)	Features: 1397832 Meta-features: 60671 Chrom/Contigs: 47	16629206
**HeLaPBS2**	36 Phred Score	22826893	20357196 (89.2%)	21859900 (95.76%)	Features: 1397832 Meta-features: 60671 Chrom/Contigs: 47	17816857
**HeLaG371**	36 Phred Score	21703211	19559128 (90.1%)	20953012 (96.54%)	Features: 1397832 Meta-features: 60671 Chrom/Contigs: 47	16515259
**HeLaG372**	36 Phred Score	23628314	20974742 (88.8%)	22699625 (96.07%)	Features: 1397832 Meta-features: 60671 Chrom/Contigs: 47	17964700
**HeLaMp1**	36 Phred Score	21224500	19155158 (90.3%)	20489226 (96.54%)	Features: 1397832 Meta-features: 60671 Chrom/Contigs: 47	16378502
**HeLaMp2**	36 Phred Score	21426871	18913666 (88.3%)	20461726 (95.50%)	Features: 1397832 Meta-features: 60671 Chrom/Contigs: 47	16264057

Supplementary Table 3: Examples of the KEGG Pathways genes affected in both conditions (Mg and Mp), as well as the leading-edge genes that drive the enrichment in each condition.

KEGG Pathway	Mg	Mp
Hematopoietic cell lineage	*TFRC, IL7R, KIT, CSF2RA, IL7,IL6*	*IL7R, IL11*
Cell adhesion molecules (CAMs)	*NRXN3, MADCAM1, MPZ, CNTN1, HLA-DOB, PDCD1LG2, PDCD1, NLGN3*	*PECAM1, CLDN7, JAM3, SELPLG*
Jak-STAT signaling pathway	*IL7R, CCND1, CSF2RA, IL7, IRF9, IL6*	*IL7R, IL11*
Neuroactive ligand-receptor interaction	*SSTR1, PTGER4, HTR1D, LTB4R, LTB4R2, GRM1, CNR1, GRM8, GCGR, TACR1, ADRB1, GPR83, PTGER1, GABRB3, PRLR*	*GRPR, OPRK1, GLRA1, GIPR, HRH4, HTR6, NPY4R, CHRNG, CHRNB2, PRSS3, GLRA3, GRIK1, CALCR, CHRNB4, GHR, HTR1F*
Insulin signaling pathway	*PPP1R3C, SH2B2, MKNK2, PCK1, PPP1R3B, HK2*	*PRKAG2*
Systemic lupus erythematosus	*H2AJ, H2AC20, HLA-DQB1, H2BU1, FCGR2A, H2AC15, H3C7, C4B, H3C14, H2AC14, H2AC21, H4C7, ACTN2, ELANE, MACROH2A2*	*H2BU1, H3C13, C4B, H3C3, H3C6, MACROH2A2, H2AC15, H3C7, FCGR2A, H3C12, C4A, HLA-DMB*
Complement and coagulation cascades	*F12, CFD, FGB, FGG, SERPINF2, C4B, CFI, PLG, SERPINA1*	*F3, SERPINE1*
Glycolysis / Gluconeogenesis	*ENO2, ALDOC, GPI, PGM1, PCK1, HK2, PGK, PFKL*	*ALDH3A1, ALDOC, PCK1*
